# Public Reporting Systems in Health Care and the Underconceptualized Technical Substrate, a Core Information Systems Dimension: Scoping Review

**DOI:** 10.2196/84820

**Published:** 2026-08-24

**Authors:** Elorm Damalie, Reima Suomi, Moustafa Mahmoud

**Affiliations:** 1 Department of Management and Entrepreneurship, Turku School of Economics, University of Turku, Rehtorinpellonkatu 3, Turku, 20540, Finland, 358 0406201515

**Keywords:** public reporting, health care quality, health care, information systems, scoping review

## Abstract

**Background:**

Public reporting systems (PRSs) in health care are defined as digital platforms that make comparative health care performance data available to the public (eg, Hospital Compare in the United States, NHS Choices in the United Kingdom, national quality registries in Europe, and MyHospital in Australia). These systems aim to improve transparency, accountability, and patient choice. While these systems have been widely studied from policy and clinical perspectives, the information system (IS) foundations that enable their operation, including architecture, interoperability, data governance, APIs, usability, and technical performance, remain underexplored.

**Objective:**

This study aims to map the extent and nature of the published literature addressing the technological foundations of PRSs in health care and to identify persistent gaps in IS scholarship.

**Methods:**

This scoping review followed the Joanna Briggs Institute guidelines and was reported in accordance with the PRISMA-ScR (Preferred Reporting Items for Systematic Reviews and Meta-Analyses Extension for Scoping Reviews). Seven databases were searched: PubMed, Web of Science, Scopus, IEEE (Institute of Electrical and Electronics Engineers), ACM (Association for Computing Machinery), AIS (Association for Information Systems) eLibrary, and Cochrane. The search covered articles published between 2000 and 2026. Eligible articles included peer-reviewed journal articles and conference papers on PRSs in health care. Data were charted according to study characteristics, including 6 IS-specific coding dimensions: architecture, interoperability, data governance, APIs, usability, and technical performance. Descriptive statistics were used to summarize the findings, and a narrative synthesis was conducted to identify thematic trends and research gaps.

**Results:**

A total of 1882 records were identified across 7 databases. After deduplication, 1127 records were screened, and 233 studies were included in the review. Most of the included studies originated from the United States (n=157, 67.4%), followed by Europe (n=43, 18.4%). Of the 233 included studies analyzed, 60.5% (n=141) used quantitative methods, whereas 24.0% (n=56) used qualitative methods and 15.5% (n=36) used mixed methods. Applying a structured IS coding framework to all 233 studies revealed that the technical substrate of PRSs remains underexplored.

**Conclusions:**

Despite the centrality of IS infrastructure to public reporting, existing scholarship predominantly frames these systems as policy or social tools while neglecting their technical underpinnings. This oversight has practical implications, including poor system usability, weak interoperability, and noncompliance with data regulations, which can undermine patient trust and system effectiveness. Addressing this gap requires interdisciplinary approaches that integrate IS frameworks, sociotechnical analysis, and usability evaluation to ensure that PRSs are effective, equitable, and technically robust.

## Introduction

In recent years, discussions on public reporting systems (PRSs) have increased due to changes in the health care sector [1,2] and the need for a consumer-centric approach to the delivery of health care [3,4]. PRSs refer to digital platforms or portals that publicly share comparative health care performance metrics [5]. Examples include Hospital Compare in the United States (publishing hospital quality indicators such as readmission and mortality rates), NHS Choices in the United Kingdom (a national portal providing performance ratings and patient experience scores), ProPublica’s Surgeon Scorecard (a journalist-led initiative reporting surgeon-specific complication rates), various national quality registries in Europe (tracking outcomes in areas such as cardiac surgery or joint replacement), and MyHospitals in Australia (reporting hospital activity and performance data) [6-11]. These platforms serve a common purpose: to enhance accountability, transparency, and patient choice [5,11,12].

Public reporting in health care started in the mid-1980s in the United States, but its roots go back to Florence Nightingale’s 1858‐1859 publication of Crimean War hospital mortality data using “coxcomb” diagrams [13]. Since then, it has been adopted and implemented in numerous countries worldwide [14,15]. These systems are designed to make comparative health care performance data accessible to a broad audience, thereby supporting consumer choice as well as provider accountability and transparency [15-17]. Public reporting is defined by Totten, Wagner [18] as “data, publicly available or available to a broad audience free of charge or at a nominal cost, about a health care structure, process, or outcome at any provider level (individual clinician, group, or organizations [e.g., hospitals, nursing facilities]) or at the health plan level” (p 3). According to Cacace et al [19], quality measurement and public reporting improve health care through 2 pathways of change: improvement through selection and improvement through change.

Improvement through selection occurs when users make their choices based on the comparative information available to them [20]. Reported comparative information enables users to make better health care decisions. This suggests that when users choose, they identify outcomes relevant to them and learn about performance levels [20].

Improvement through change is a pathway in which health care providers improve their services based on published comparative data [20]. For example, a hospital can improve postoperative care to reduce infection rates. This type of improvement originates with the provider, thereby improving the quality of care received by users. The user is the main beneficiary of both pathways of change, as each leads to improved quality of health care services.

According to Werner and Asch [21], public reporting has been associated with a small decline in mortality rates. There have been reported cases of the positive effects of public reporting on health care centers and providers [22]. Werner and Asch [21] report on the impact of public reporting on quality improvement interventions in nursing homes. The findings of this study [4] indicate that the public reporting of performance data has led health care organizations to improve and maintain high levels of performance.

According to research, one of the benefits of public reporting is the freedom of choice for consumers [23]. Freedom of choice in health care refers to consumers deciding on and selecting their own health care providers [24,25]. According to Zolkefli [25], this enhances consumer autonomy. This is a consumer-centric approach to health care delivery. The need for freedom of choice stems from the fact that the more choices consumers have, the greater their freedom and autonomy, which in turn leads to greater well-being and a sense of personal worth [25,26].

The growing role of PRSs in health care through information systems (ISs) and data use has fostered consumer or patient centrism in health care [27]. ISs, including digital platforms, big data analytics, and cloud technologies, play a significant role in providing health care information to patients and facilitating information sharing between providers and patients [28-30]. As a result, patients now have greater access to both quantitative and qualitative health care information, enabling more informed decision-making [31,32]. This has led to the development of digital platforms such as PRSs, which provide information to patients and the public.

Previous reviews have examined PRSs in health care settings [5,18,33], but to the best of our knowledge, no review to date has mapped public reporting from the viewpoint of studying the role, design, development, and use of ISs as an infrastructural cornerstone of public reporting. This review offers valuable insights for practitioners and researchers in ISs. It also provides a cross-disciplinary synthesis for health care policymakers and digital health stakeholders by integrating insights from the public health, informatics, and IS domains. It provides a roadmap that pinpoints what is already known, what remains unexamined, and where IS experts can intervene to build, evaluate, or refine the digital infrastructures underpinning PRSs [34-36].

While related domains such as patient portals, clinical quality registries, and digital dashboards have received attention in health informatics and ISs [37-43], these typically emphasize clinical outcomes or patient engagement in narrower contexts. What remains underexplored is the technological substrate specific to PRSs: how architectures are designed [44], interoperability standards (eg, Health Level Seven International [HL7] or Fast Healthcare Interoperability Resources [FHIR]) are implemented [45,46], data governance is operationalized [47], and usability is evaluated at scale [48-50]. This distinction motivates our scoping review and justifies treating public reporting as a distinct IS artifact rather than a generic health IT application.

This scoping review aims to map the extent to which the published literature addresses the technological foundations of PRSs and to identify the persisting gaps in IS scholarship. This scoping review is guided by the research question: *What research exists regarding the role of information systems in facilitating public reporting systems within health care?* The objective is not to evaluate the technical quality of existing systems but rather to map the attention given to technological dimensions in the literature, thereby pinpointing areas that have yet to receive attention from IS researchers. The review proposes recommendations for advancing research in public reporting from the IS perspective.

## Methods

### Design and Rationale

Scoping reviews are conducted to identify and map evidence that is available on a specific topic [34]. Munn et al [51] provide a detailed definition of a scoping review: “Scoping reviews are a type of evidence synthesis that aims to systematically identify and map the breadth of evidence available on a particular topic, field, concept, or issue, often irrespective of source (i.e., primary research, reviews, non-empirical evidence) within or across particular contexts. Scoping reviews can clarify key concepts/definitions in the literature and identify key characteristics or factors related to a concept, including those related to methodological research” (p 950). Based on this definition, scoping reviews are important for understanding the existing body of evidence on a topic and shaping the direction of future research. The underlying difference between a scoping review and a systematic review is that scoping reviews have a broader scope and differ in their primary purpose [52]. A scoping review is necessary to explore the nature and status of PRSs in health care, as this research field remains scarcely mapped.

This scoping review was conducted according to the Joanna Briggs Institute (JBI) guidance [53] for scoping reviews and is reported in line with the PRISMA-ScR (Preferred Reporting Items for Systematic Reviews and Meta-Analyses Extension for Scoping Reviews; Checklist 1) [36]. The steps in this scoping review are documented below.

### Protocol and Registration

The protocol was registered on the Open Science Framework on July 8, 2025 (Open Science Framework registration: osf.io/mnc8x), and updated on April 3, 2026, to enhance transparency and provide a permanent record of methods. Although prospective registration is preferable, no deviations from the prespecified eligibility, screening, data charting, or synthesis methods occurred, consistent with JBI and PRISMA-ScR guidance.

### Eligibility Criteria (Population, Concept, and Context Framework)

The Population, Concept, and Context framework guiding eligibility in this review is summarized in Table 1.

**Table 1.** Population, Concept, and Context (PCC) framework.

PCC element	Operational definition used in this review
Population	Any health service provider or health systems that make comparative performance information publicly available.
Concept	Public reporting of health care quality or performance, including systems that provide comparative information for public access.
Context	All countries, all levels of care (primary, secondary, tertiary, long term), and all payer or ownership models. No setting restrictions.

### Inclusion and Exclusion Criteria

The inclusion and exclusion criteria applied in this study are detailed in Textbox 1.

We limited inclusion to English-language publications because most of the indexed work in this domain is in English. We also limited the timeframe to January 1, 2000, to February 4, 2026, to capture the modern era of web-based public reporting and contemporary health information standards [54-56].

Textbox 1. **Inclusion and exclusion criteria.**

**Inclusion criteria**

- English language
- Published between January 1, 2000, and February 4, 2026
- Papers that describe, design, or evaluate
- Any source describing real-world or pilot public reporting tools

**Exclusion criteria**

- Reporting designed solely for internal quality improvement, audit surveillance, or regulatory compliance
- Health care is mentioned, but the article does not study reporting
- Gray literature
- Editorials, protocols, dissertations, not accessible in full texts

### Calibration and Reliability

Two reviewers (ED, MM) piloted the screening of 50 records to calibrate the application of the eligibility criteria. Disagreements were discussed, and the decision rules were refined. Agreement during the pilot was substantial (Cohen *k*=0.74 for title or abstract, *k*=0.81 for full text). Following calibration, we proceeded with dual-independent screening of all remaining records. Disagreements were resolved by consensus with a third reviewer (RS) when required.

### Rationale for Timeframe

The rationale for the January 1, 2000, to February 4, 2026 timeframe is that since the early 2000s, public reporting has progressed from static consumer-oriented portals to interactive dashboards, integrated reporting platforms, and interoperability-focused systems. This reflects the evolution of the IS infrastructure underpinning public reporting.

### Information Sources and Search Strategy

A comprehensive literature search was conducted across 7 databases with the assistance of an academic librarian. The databases were ACM (Association for Computing Machinery), IEEE (Institute of Electrical and Electronics Engineers), Web of Science, PubMed, Scopus, Cochrane (Cochrane Collaboration), and AIS (Association for Information Systems) eLibrary. These 7 databases were chosen because of the interdisciplinary nature of the research question. Public reporting spans both health care and ISs, which shaped the decision to search databases from both domains. The search strategy was developed with an academic librarian, using keywords and synonyms related to public reporting (eg, “public performance,” “report card,” “quality report”) combined with “healthcare” using Boolean operators. The second strand was an IS-targeted search, adding technical synonyms including “architecture,” “interoperability,” “FHIR,” “HL7,” “data governance,” “application programming interface (API),” “usability,” “user experience,” “dashboard,” and “platform,” combined with the public reporting terms. Full search strings for all 7 databases are provided in Multimedia Appendix 1. The search was carried out across titles, abstracts, and keywords. A total of 1882 articles were retrieved and imported into the EndNote reference management software for further analysis. Reviewers (ED, MM) removed duplicates before conducting further screening.

### Data Charting

An extraction spreadsheet for charting was developed in Microsoft Excel to extract the relevant information needed to address the objectives of this review. The data extraction was piloted on 50 articles included in this review, and all modifications were made based on discussions and feedback from the reviewers, in an iterative process. The modifications included the addition of new columns for extracting the aim of the study and the results of all 233 articles.

All 1127 records were manually screened by 2 independent reviewers (ED and MM) in 2 stages (title or abstract screening and full-text screening). The final set of 233 included articles was read in full and manually charted by the reviewers (ED and MM), ensuring comprehensive coverage of the evidence base. Discrepancies at each stage were resolved through consensus, with RS acting as a third reviewer when required.

The complete extraction table, containing study-level details for all 233 included articles, is provided in Multimedia Appendix 2.

### Data Items

The data items included the following:

- Authors
- Affiliation
- Country
- Title
- Year
- Journal
- Type of study
- PRS type
- Method
- Sample size
- Main outcome
- Field
- Architecture
- Interoperability
- Data governance
- APIs
- Usability
- Technical performance
- Quantitative, qualitative, or mixed method

### IS Coding Framework and Reliability

Each of the 6 IS dimensions was coded using the following categories: absent (the study explicitly indicated that the dimension was absent), limited (the dimension was mentioned briefly without technical detail), moderate (the dimension was described with some technical or operational detail), strong (the dimension was examined systematically with substantial technical detail), and not reported (the study did not provide sufficient information to assess the dimension). For borderline cases, hybrid categories (limited/moderate and moderate/strong) were used. Reviewers also retained qualitative justification notes for each classification. Interrater reliability for the IS coding dimensions ranged from 0.71 to 1.00 (Cohen *k*), indicating substantial to almost perfect agreement.

### Data Analysis and Presentation

Descriptive statistics (frequencies, percentages, and graphs) were used to summarize study characteristics including geographic distribution, study design, and publication trends. Nominal data were presented in tables and figures. Narrative synthesis was used to identify overarching themes, interpret the quantitative coding findings, and identify research gaps.

### Critical Appraisal

Consistent with the JBI guidance for scoping reviews, we did not undertake a formal risk-of-bias appraisal because the objective of a scoping review is to map the breadth and nature of the evidence rather than to synthesize effectiveness or causality.

## Results

### Study Selection

**Figure 1.**
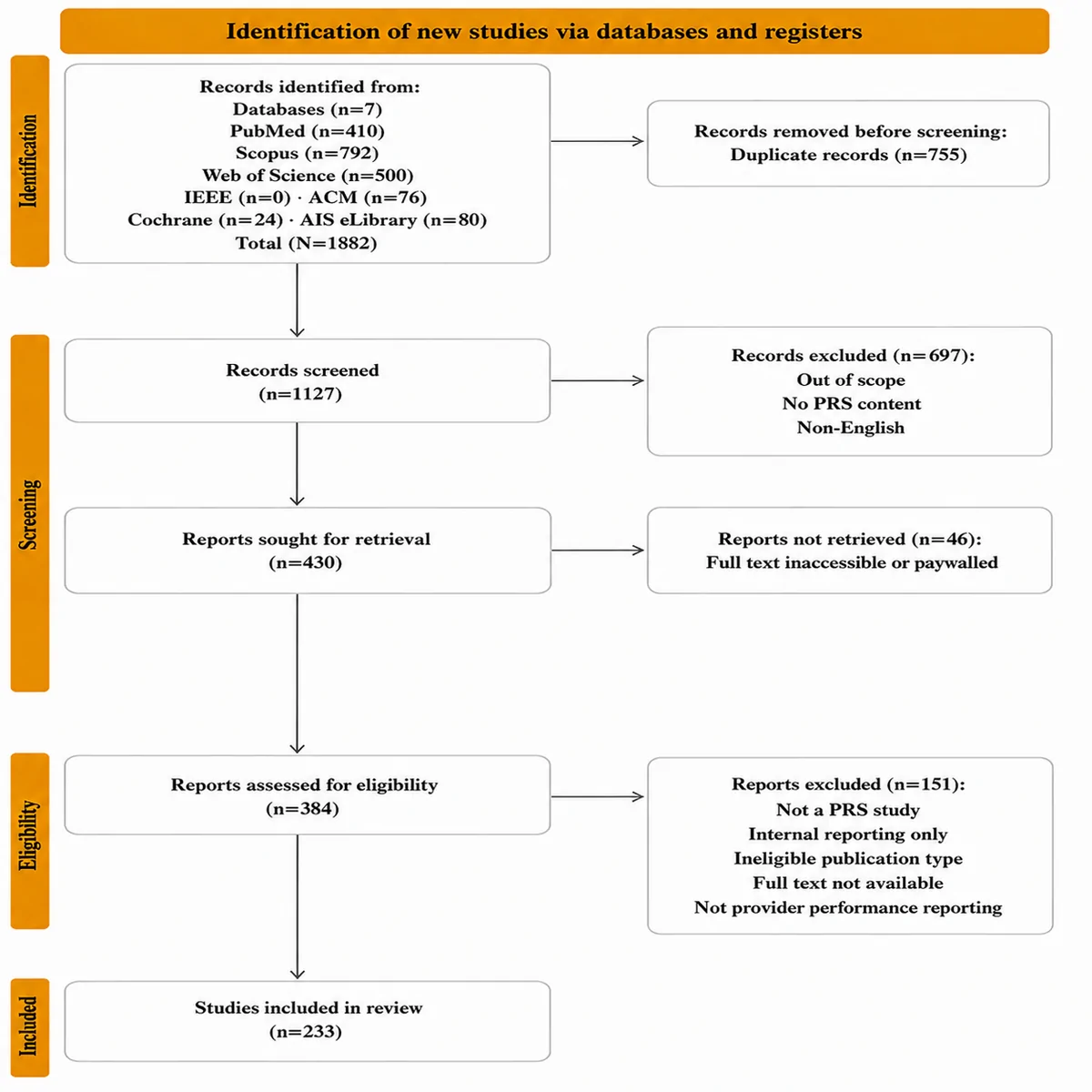
PRISMA-ScR (Preferred Reporting Items for Systematic Reviews and Meta-Analyses for Scoping Reviews) flow diagram of the search and study selection process. ACM: Association for Computing Machinery; AIS: Association of Information Systems; IEEE: Institute of Electrical and Electronics Engineering; PRS: public reporting system.

The database search yielded 1882 articles across 7 databases: PubMed (n=410), Scopus (n=792), Web of Science (n=500), IEEE (n=0), ACM (n=76), Cochrane (n=24), and the AIS eLibrary (n=80). All articles were imported into Endnote, where 755 duplicate articles were identified and removed, leaving 1127 unique records for title, abstract, and keyword screening. Two reviewers (ED and MM) independently screened 1127 articles against the inclusion criteria listed in Textbox 1. Conflicts were resolved by consensus. Of these, 697 articles were excluded, leaving 430 articles for phase 2 of full-text screening against the exclusion criteria. The same reviewers (ED and MM) independently screened 430 articles for full-text retrieval, of which 46 could not be obtained due to inaccessible or paywalled full texts. The 384 retrieved articles were independently assessed for eligibility by the same 2 reviewers. A further 151 articles were excluded at full-text review for the following reasons: not a PRS study, reporting designed solely for internal quality improvement purposes, ineligible publication type, full text not available upon further attempts, or not concerned with provider performance reporting. Disagreements at both screening stages were resolved through discussion, with a third reviewer (RS) acting as arbitrator where consensus could not be reached. A final corpus of 233 articles was included in the review. The full study selection process is presented in the PRISMA-ScR flow chart in Figure 1.

### Characteristics of Included Articles

The characteristics of the included articles in this scoping review are reported in Tables 2 and 3. The 233 included articles span 18 countries and settings (Table 2). The United States dominates the literature, accounting for 157 articles (67.4% of all included studies), reflecting the prominence of US-based PRSs such as Hospital Compare, Nursing Home Compare, and the Centers for Medicare & Medicaid Services (CMS) quality programs. Germany is the second most represented country, with 14 (6.0%) articles, followed by multicountry or international studies, also with 14 (6.0%) articles, which include systematic reviews, cross-national comparisons, and studies covering multiple high-income countries. The United Kingdom contributes 12 (5.2%) articles, with studies drawing primarily on NHS and Hospital Episode Statistics data. Australia and the Netherlands each account for 7 (3.0%) articles, and Canada accounts for 6 (2.6%) articles. Norway contributes 3 (1.3%) articles, while Japan, France, and China each have 2 (0.9%) articles. The remaining countries—Tajikistan, Belgium, Republic of Korea, Switzerland, Sweden, Italy, and Finland—each contribute a single (0.4%) article, reflecting emerging but limited international scholarship on PRSs outside North America and Western Europe.

**Table 2.** Geographic distribution of the 233 included studies by country (n=233).

Countries	Studies, n (%)
United States	157 (67.4)
Germany	14 (6.0)
Multicountry	14 (6.0)
United Kingdom	12 (5.2)
Netherlands	7 (3.0)
Australia	7 (3.0)
Canada	6 (2.6)
Norway	3 (1.3)
Japan	2 (0.9)
France	2 (0.9)
China	2 (0.9)
Tajikistan	1 (0.4)
Belgium	1 (0.4)
Republic of Korea	1 (0.4)
Switzerland	1 (0.4)
Sweden	1 (0.4)
Finland	1 (0.4)
Italy	1 (0.4)

**Table 3.** Methodological characteristics of the 233 included studies.

Study design	Studies, n (%)
Quantitative	141 (60.5)
Qualitative	56 (24.0)
Mixed methods	36 (15.5)

### Study Design

The included articles reflect a predominantly quantitative literature (Table 3). The largest group comprises quantitative observational studies (n=100, 42.9%), encompassing cohort studies, cross-sectional analyses, retrospective database studies, and longitudinal evaluations of publicly reported quality metrics. The second-largest group comprises qualitative, conceptual, and review studies (n=56, 24.0%), including systematic reviews, narrative reviews, policy analyses, conceptual papers, and qualitative interview studies examining stakeholder perceptions and implementation experiences. Mixed methods studies account for 36 (15.5%) articles, combining qualitative and quantitative approaches. Methodological and validation studies represent 27 (11.6%) articles, focusing on statistical methods such as risk adjustment, profiling methods, measure development, and simulation studies. Finally, quantitative survey, experimental, and cohort studies make up 14 (6.0%) articles, including randomized web experiments, online surveys, and choice experiments examining consumer responses to publicly reported information.

**Figure 2.**
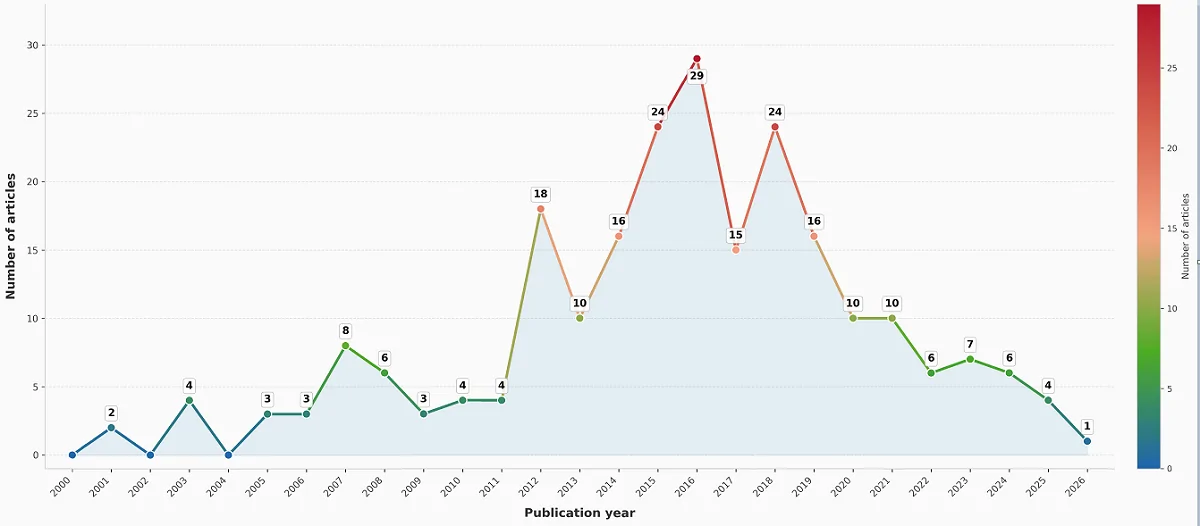
Annual distribution of included studies by publication year (2000‐2026)*.*

Figure 2 shows a clear trend in publications on PRSs, rising steadily from 2001 to 2016, followed by a gradual decline (Multimedia Appendix 3). Output was sparse in the early 2000s, averaging fewer than 5 studies per year prior to 2012, with a brief exception in 2007-2008. A sharp increase began around 2012 (n=18), reaching a peak in 2016 (n=29, 12.4%). After 2016, publication volume declined but remained relatively active through 2019 to 2021, with 16 studies in 2019 and 10 studies in each of 2020 and 2021. Output tapered further from 2022 onward, with only 1 study recorded in 2026, reflecting the early search date. Overall, the bulk of the literature was published between 2012 and 2019, accounting for more than half of all included articles.

### IS Dimension Coverage

The central quantitative finding of this review is summarized in Table 4, which presents the distribution of IS coding levels across all 6 dimensions for the 233 included studies.

**Table 4.** Information systems dimension coverage across the 233 included studies, assessed using a structured coding framework comprising 6 core technical substrate dimensions^a^.

Dimension	Absent, n (%)	Limited, n (%)	Limited/moderate, n (%)	Moderate, n (%)	Moderate/strong, n (%)	Strong, n (%)	Not reported, n (%)
Architecture	—^b^	69 (29.6)	1 (0.4)	35 (15.0)	—	16 (6.9)	112 (48.1)
Interoperability	—	60 (25.8)	1 (0.4)	34 (14.6)	1 (0.4)	14 (6.0)	123 (52.8)
Data governance	—	30 (12.9)	—	76 (32.6)	2 (0.9)	115 (49.4)	10 (4.3)
APIs^c^	9 (3.9)	—	—	—	—	1 (0.4)	223 (95.7)
Usability	—	78 (33.5)	3 (1.3)	52 (22.3)	4 (1.7)	38 (16.3)	58 (24.9)
Technical performance	—	59 (25.3)	5 (2.1)	69 (29.6)	1 (0.4)	93 (39.9)	6 (2.6)

a Absent: the study explicitly indicated that dimension was absent; limited: the dimension was mentioned briefly without technical detail; moderate: the dimension was described with some technical or operational detail; strong: the dimension was examined systematically with substantial technical detail; not reported: insufficient information was provided to assess the dimension. Hybrid categories (limited/moderate and moderate/strong) were used for borderline cases, and qualitative justification notes were retained in the extraction table.

b Not available.

c API: application programming interface.

#### Architecture

Architecture received no ISs relevant coding in 48.1% (n=112, not reported) of the studies and was rated limited or limited or moderate in a further 30.0% (n=70) of the studies, meaning 78.1% (n=182) of the included studies provide no substantive IS analysis of system architecture. Moderate architecture coverage was identified in 15.0% (n=35) of the studies. Strong architecture coverage was identified in only 16 (6.9%) studies. Exemplars include Cebul [57], describing an electronic medical record–integrated diabetes registry with explicit infrastructure detail (strong architecture, strong data governance, strong technical performance), Fu et al [58] demonstrate health information exchange–based automated quality reporting through the Long Beach Network for Health, and Friedrichson et al [59] describe an interactive national Extracorporeal Membrane Oxygenation dashboard built from merged public reimbursement data and hospital quality reports. These studies share a common feature: they were authored by teams that include IS or informatics researchers alongside clinical domain experts, a collaboration pattern absent from the vast majority of the corpus.

#### Interoperability

Interoperability received no IS relevant coding in 52.8% (n=123, not reported) of the studies and was rated limited or limited or moderate in a further 26.2% (n=61) of the studies. Collectively, 79.0% (n=184) of the included studies provided no substantive engagement with interoperability standards. Strong interoperability coverage was found in only 14 (6.0%) studies. No study systematically evaluated FHIR-based exchange for PRS, and only 2 studies mentioned HL7 standards in any substantive technical sense. Mentions of “data sharing” or “online reporting portals” without reference to interoperability standards were common and were coded as absent. Studies with moderate interoperability typically described registry-to-claims data linkage processes; those with strong ratings (n=14) included national-scale registries with documented data integration pipelines.

#### Data Governance

Data governance was the strongest-performing dimension: 49.4% (n=115) of studies were rated strong, and 32.6% (n=76) were rated moderate, with a further 0.9% (n=2) rated moderate or strong. However, a critical finding concerns the nature of this governance coverage. Detailed examination reveals that strong and moderate governance ratings in this corpus almost exclusively reflect measurement governance, including risk adjustment methodology, measure validity, reliability testing, indicator construction rules, and statistical quality controls. Data stewardship governance, including data provenance tracking, access control frameworks, consent management, and alignment with regulatory frameworks such as General Data Protection Regulation or Health Insurance Portability and Accountability Act, was effectively absent. For example, measurement governance determined whether a hospital mortality indicator uses a 30-day or 90-day follow-up period and how risk adjustment is specified [60,61]; infrastructure data governance determines how source data are transferred, secured, versioned, and traced through the reporting pipeline [62-64]. This conflation between measurement validity and system-level data governance is a recurring conceptual error with implications for how PRSs evidence translates into system design.

#### APIs

APIs represent the most acute gap in the PRS literature. Nearly all studies (232, 99.6%) contained no discussion of APIs, open data end points, or programmatic data access. The single exception, Fife et al [65], described a wound registry-based Qualified Clinical Data Registry under Merit-based Incentive Payment System, mentioned open API support only as a future aspiration rather than an implemented technical feature. This finding is particularly significant given that the 21st Century Cures Act (2016, enacted 2021) and the CMS Interoperability and Patient Access Final Rule (2020) have made FHIR-based, open API access a regulatory requirement for health IT systems in the United States, the source of 67.4% (n=157) of this corpus. The literature has simply not registered this regulatory infrastructure shift.

#### Usability

Usability evidence was present but systematically skewed. Strong usability was identified in 16.3% (n=38) of the studies, with most of it concentrated in consumer-facing format experiments: icon versus numerical display, star ratings versus word labels, narrative versus structured performance data, and health literacy-focused accessibility work. These studies collectively constitute a well-developed subliterature on how patients read and respond to publicly reported data. By contrast, the usability of provider-facing data submission interfaces, administrator-facing reporting dashboards, and the data collection workflows that generate PRS data was almost entirely absent. The asymmetry is a structural gap in the field: rich knowledge of what patients see, near-zero knowledge of the systems that produce what they see.

#### Technical Performance

Technical performance was rated strong in 39.9% (n=93) of the studies and moderate in a further 29.6% (n=69) of the studies, but detailed coding revealed that this predominantly reflected statistical performance rather than IS system performance. C-statistics, calibration curves, model discrimination, receiver operating characteristic analyses, and measurement reliability coefficients, all statistical properties of measurement instruments, were routinely reported under what we initially coded as technical performance. Once IS performance (latency, throughput, scalability, uptime, data refresh frequency) was recorded separately from statistical performance, fewer than 8% (n=18) of the studies contained genuine IS system performance data. This conflation of measurement validity with IS performance pervaded the literature, representing a systematic miscategorization of what is technically known about PRS infrastructure.

## Discussion

This scoping review mapped 233 studies on PRSs in health care published between 2000 and 2026. Applying a technical substrate framework, which is a core dimension of IS comprising architecture, interoperability, data governance, APIs, usability, and technical performance to all included studies, reveals a pattern of coverage that is both uneven and shallow in analytical depth.

### Technical Substrate: A Core IS Dimension That Is Systematically Absent

The technical substrate, one of the core IS dimensions through which PRSs can be analyzed, is unevenly and sparsely represented across the 233 included studies. This is important because IS research has long argued that system quality, information quality, infrastructure, and the IT artifact itself should not be treated as neutral or invisible background conditions but as central objects of analysis [66-68]. Architecture received substantive IS engagement (moderate or strong) in only 21.9% (n=51) of the studies, interoperability was engaged at a strong level in only 6.0% (n=14) of the studies, and usability was addressed at a strong level in 16.3% (n=38) of the studies, though almost exclusively in consumer-facing format experiments rather than system-level evaluations.

Where technical substrate elements appeared, they were incidental rather than operationalized and were referenced without architectural specification, formal testing, or analytical depth. These are not marginal omissions. PRSs are, at their core, large-scale ISs that collect, aggregate, standardize, validate, and disseminate sensitive health care data across heterogeneous providers, administrative systems, and public interfaces. The design choices embedded in their architecture determine what data are collected, how data are transformed, who can access them, and whether the resulting output is trustworthy and actionable. Health informatics research makes clear that secondary use of health care data requires explicit attention to data quality dimensions such as completeness, correctness, concordance, plausibility, and currency, as well as harmonized terminology, stewardship, and governance practices [69-71].

The absence of IS scholarship in this domain means that those design choices are being made without the theoretical or empirical guidance that IS research could provide. Without examining the technical substrate, stakeholders cannot generate actionable guidance for designing interoperable, trustworthy, user-centered reporting systems, which limits the capacity of public reporting to improve care quality and patient empowerment despite significant public investment.

### Data Governance: Conflation of Indicator Quality With Infrastructure Governance

A critical interpretive finding concerns the nature of data governance coverage. On the surface, data governance appears to be a relative strength, with 82.8% (n=193) of the studies rated moderate or strong. However, detailed examination reveals that these ratings almost exclusively reflect measurement governance, including the validity, reliability, and statistical properties of performance indicators [72,73], rather than system-level data governance such as data provenance frameworks, access control architectures, consent management, or alignment with regulatory requirements such as the General Data Protection Regulation or the Health Insurance Portability and Accountability Act [70,74-77]. This conflation between indicator quality and infrastructure governance is not merely a semantic distinction; it represents a fundamental gap in the field’s understanding of what makes PRSs trustworthy, secure, and sustainable. No included study examined how rules and responsibilities for data stewardship, security, and accountability are formally designed in PRSs. This is a gap with urgent practical implications for compliance risk and public trust.

### Usability: Consumer-Facing Coverage and Provider-Facing Invisibility

A further structural asymmetry was identified in usability coverage. Consumer-facing usability constitutes a relatively well-developed subliterature, covering how patients read star ratings, navigate comparative dashboards, or interpret numerical versus icon-based displays, with strong usability identified in 16.3% (n=38) of the studies, concentrated in format experiments.

By contrast, the usability of provider-facing data submission interfaces, administrator-facing dashboards, and the data collection workflows that generate public reporting data is virtually absent. The systems that produce what patients see are, from a usability perspective, invisible in the literature. This absence is important because health care IS research has repeatedly shown that usability must be studied with the full sociotechnical work system, including users, tasks, tools, workflows, organizations, and the wider environment, rather than only at the final user-facing display layer [78-80].

This asymmetry has direct practical consequences: poorly designed provider-facing interfaces increase reporting burden, introduce data entry errors, and undermine the quality of the very outputs that consumer-facing usability studies seek to improve. An IS-informed approach to PRSs must address both ends of the data pipeline. This means evaluating not only the public interface through which patients compare providers, but also the upstream processes through which data are entered, validated, aggregated, transformed, and released. Such an approach aligns with IS success theory, which treats system quality, information quality, use, user satisfaction, and net benefits as interdependent, and with sociotechnical health IT research, which emphasizes that system outcomes depend on the interaction between technical design and organizational work practices [66,80].

### Geographic Concentration and the Regulatory Evidence Gap

Geographically, 67.4% (n=157) of the included studies originate from the United States, yet the regulatory infrastructure shaping US health data interoperability, including the 21st Century Cures Act (2016/2021) [81] and the CMS Interoperability and Patient Access Final Rule (2020) [82], which mandate FHIR-based open API access for health IT systems, is simply not reflected in the literature.

The corpus has not registered this regulatory shift, leaving a significant and growing gap between regulatory requirements and research evidence. This is consistent with the broader Global North dominance in the corpus. Europe ranked second at 18.4% (n=43), led by Germany and the United Kingdom. This distribution underscores an important limitation: contexts with constrained digital infrastructures or limited regulatory capacity face unique challenges in implementing PRSs, yet these challenges remain underexplored. Representation from Asia was limited, while Africa and South America were not represented in the included studies. This is a notable underrepresentation of low- and middle-income countries.

### Structural Drivers of the IS Gap

The disciplinary absence of IS scholarship in PRS research appears to reflect structural features of the field rather than a merely accidental omission. Three drivers may help explain this pattern. First, PRS research is predominantly published in health services research, public health, and health policy journals whose editorial cultures do not include IS expertise, creating a self-reinforcing publication environment [83-85]. Second, health care research funders historically prioritize clinical outcomes and health services effectiveness over system design: infrastructure expenditure for PRSs is absorbed into procurement budgets without generating peer-reviewed IS scholarship.

Third, the dominant paradigm in PRS research concerns whether public reporting changes behavior, including patient choice, provider performance, reputation, or market share, rather than how the IS enabling it is designed, maintained, or improved. This reflects a broader tendency in health policy research to treat digital infrastructure as a background condition rather than an object of inquiry in its own right [3,80,86].

These findings should not be read as an argument for technological solutionism. Sociopolitical factors, including provider resistance, regulatory constraints, patient health literacy, and health care market structures, shape the effectiveness of PRSs in ways that improved system architecture alone cannot resolve. The IS contribution is to make these technical dimensions legible, improvable, and empirically tractable, not to claim that they are determinative of outcomes.

### Comparison to Prior Work

Prior scoping reviews and systematic reviews of public reporting have concentrated on its policy effects: whether public reporting changes provider behavior, reduces mortality, improves patient experience, or influences hospital choice. Fung et al [33], Campanella et al [22], and Cacace and Geraedt [5] established the evidentiary landscape for these questions. This review does not duplicate that work but adds a complementary dimension: it asks not whether public reporting works but whether the ISs that deliver it are being studied adequately.

This review is the first, to our knowledge, to apply a systematic IS coding framework to the public reporting literature and to demonstrate, with quantitative evidence rather than narrative assertion, that the architectural, interoperability, governance, and API dimensions are largely invisible in the research corpus.

Adjacent literatures are more advanced: patient portal usability has been well studied, and electronic health record interoperability has received sustained informatics attention. PRSs share infrastructure with all 3 but have not benefited from the IS analysis that those adjacent domains have received.

The exemplar studies identified in this review, including Cebul [57], Fu et al [58], Friedrichson et al [59], and Dehmer et al [87], demonstrate that IS-informed public reporting research is feasible and productive. These studies were consistently authored by teams that include IS or informatics researchers and clinical domain experts. Their scarcity underlines the structural driver of the IS gap: it is not that PRSs are technically unanalyzable, but that the research communities studying public reporting and those studying health ISs have remained largely separate.

### Limitations

First, although we added a supplementary IS targeted search strand and expanded to 7 databases including the AIS eLibrary, it remains possible that IS native literature using heterogeneous terminology was not fully captured. Future reviews should refine the dual-strand approach and consider forward citation searching from the 5 exemplary studies identified here.

Second, our IS coding involved judgments about coverage level that are inherently interpretive. Interrater reliability was substantial to nearly perfect across dimensions (Cohen *k* range 0.71‐1.00, as reported in the *Methods* section), but residual subjectivity cannot be eliminated, particularly for the distinction between limited and moderate.

Third, the corpus is dominated by literature from the United States (n=157, 67.4%), which limits generalizability to health care systems with different IS infrastructure and regulatory contexts. Low- and middle-income country settings, where PRSs may be developing on entirely different technical foundations (mobile-first, low-bandwidth architectures), are almost entirely absent.

Fourth, as this was a scoping review conducted per JBI guidance, formal critical appraisal was not performed.

Fifth, although the inclusion period extended to February 4, 2026, the searches were conducted early in the year. Publications from later in 2026 are therefore not represented, which may limit the review’s ability to capture the most recent regulatory and interoperability-related developments, including the ongoing implementation of the 21st Century Cures Act and FHIR-based interoperability mandates.

### Recommendations for Future Research

We identified several critical gaps in the literature on PRSs, with direct implications for practice. First, the included studies show a significant lack of research on the technical substrates, a core IS dimension of PRSs. Yet, these technical foundations, namely architecture, interoperability, APIs, technical performance, and data governance, are precisely what make PRSs feasible in practice. More research is therefore needed on the technical substrates of PRSs, especially because these systems function as information platforms for patients, providers, policymakers, and the public.

Second, future research should investigate data enhancement for PRSs in health care. Improvements in data quality, completeness, timeliness, and usability are directly linked to the quality of information presented through PRSs. Third, the role of API integration is notably absent from current studies, despite its practical importance. Without robust APIs and interoperable standards, health systems risk perpetuating data silos and increasing the reporting burden on providers. Research should therefore investigate how integration can streamline data flows and enable scalable, efficient, and user-friendly reporting infrastructure.

Fourth, data governance is essential in any data-intensive system. PRSs are data-driven, yet no included study examined how rules and responsibilities for data stewardship, security, accountability, and privacy are designed in these systems. This gap has urgent practical implications. Without strong governance, organizations face compliance risks, privacy breaches, reduced data integrity, and erosion of public trust. Future research should therefore examine how data governance frameworks can safeguard both data quality and public confidence.

Finally, qualitative multiple case research should be used to examine PRSs across different national and organizational contexts. This approach would allow an in-depth exploration of how institutions, policy environments, technical infrastructures, and stakeholder expectations affect the adoption and implementation of PRSs. It would also support the identification of implementation challenges and best practices, offering practical guidance for governments and health organizations seeking to design PRSs that work in real-world conditions.

A comprehensive understanding and effective use of PRSs require an integrated evidence base that spans technical, organizational, and policy dimensions. By highlighting the practical risks of overlooking the technical substrate, this review underscores the need to realign future research priorities. In particular, future scholarship should incorporate IS research more directly into the study of PRSs and generate actionable insights to design reliable, interoperable, scalable, and equitable public reporting infrastructures.

Based on our findings, we propose 6 specific IS focused research questions (RQs):

- RQ1: Architecture: What technical IS architectures (centralized vs federated registry; FHIR-native vs claims-based) are most effective for enabling accurate, timely, and scalable PRS data pipelines, and what implementation trade-offs arise across different health care system types?
- RQ2: APIs and interoperability: What API architectures (Representational State Transfer, Fast Healthcare Interoperability Resources, Release 4, Substitutable Medical Applications and Reusable Technologies on FHIR, clinical decision support Hooks) are most effective for enabling real-time, automated data submission to national PRS platforms, and what is their relative implementation burden for small versus large provider organizations?
- RQ3: FHIR adoption effects: How does the adoption of HL7 FHIR-based interoperability standards affect data acquisition cost, timeliness, completeness, and accuracy in national quality registries, compared with traditional administrative claims-based approaches?
- RQ4: IS success model applied to PRS: Using the DeLone and McLean IS success model as a theoretical lens, which IS quality dimensions (information quality, system quality, service quality) are most predictive of the impact of PRSs on provider behavior change and measurable quality improvement?
- RQ5: Data governance: How are data governance responsibilities, including stewardship, accountability, privacy, security, and compliance, designed and implemented in PRSs, and how do these governance arrangements affect data integrity, public trust, and system sustainability?
- RQ6: Comparative sociotechnical implementation: How do institutional, policy, organizational, and technological factors shape the adoption and implementation of PRSs across different countries and health care settings, and what best practices can be identified through qualitative multiple case research?

### Conclusions

This scoping review maps research on PRSs in health care published between January 2000 and February 2026 and provides, to our knowledge, the first systematic quantitative characterization of the extent to which the technical substrate of PRSs, comprising architecture, interoperability, data governance, APIs, usability, and technical performance, has been addressed in the published literature. Across 233 studies, APIs were absent or not reported in 99.6% (n=232) of the studies. Architecture was not reported, limited, or limited or moderate in 78.1% (n=182) of the studies, while interoperability was not reported, limited, or limited or moderate in 79.0% (n=184) of the studies. These gaps are not merely academic. PRSs that lack interoperable, API-enabled infrastructure are unlikely to be effectively integrated into clinical workflows, updated in real time, or queried by third-party developers. Similarly, platforms without explicit data stewardship governance are vulnerable to provenance failures, reidentification risks, and regulatory noncompliance. Platforms whose provider-facing usability has never been studied may impose unmeasured reporting burdens that compromise data quality and erode clinical trust.

Advancing the field requires interdisciplinary collaboration between health care outcomes researchers, health informatics scholars, IS scientists, and the practitioners who build and maintain reporting systems. The 6 research questions proposed here offer a traceable starting point. The 4 exemplary studies identified, spanning electronic medical record–integrated diabetes registry, health information exchange–based automated reporting, national Extracorporeal Membrane Oxygenation dashboard, and national clinical dashboards, offer architectural templates. The DeLone and McLean IS success model within sociotechnical systems perspectives provides a conceptual scaffold. Without systemic examination of the technical substrate, which is a core dimension of ISs, PRSs risk becoming well-intentioned tools that deliver incomplete or inaccessible data, frustrate patients with poor usability, and burden providers with fragmented noninteroperable platforms. As a result, they may fall short of becoming the reliable, equitable, and technically robust digital infrastructures that genuine health care transparency demands.

## Supplementary material

10.2196/84820 Multimedia Appendix 1 Complete search strings (all 7 databases).

10.2196/84820 Multimedia Appendix 2 Extraction table.

10.2196/84820 Multimedia Appendix 3 Annual distribution of included studies by publication year (2000-2026).

10.2196/84820 Checklist 1 PRISMA-ScR checklist.

## Abbreviations

ACM: Association for Computing Machinery
AIS: Association for Information Systems
CMS: Centers for Medicare & Medicaid Services
FHIR: Fast Healthcare Interoperability Resources
HIE: health information exchange
HL7: Health Level Seven International
IS: information system
JBI: Joanna Briggs Institute
PRISMA-ScR: Preferred Reporting Items for Systematic Reviews and Meta-Analyses extension of Scoping Reviews
PRS: public reporting system
RQ: research question
