# Supplementary material for: Public Reporting Systems in Health Care and the Underconceptualized Technical Substrate, a Core Information Systems Dimension: Scoping Review
Source: JMIR Med Inform. 2026 Aug 24;14:e84820. doi: 10.2196/84820 (PMC13502770; doi:10.2196/84820)
Supplement: Supplementary file 1 [file medinform-v14-e84820-s001.docx]

**Database search queries**

| Date | Database | Search String |
| --- | --- | --- |
| 4.2.2026 | Pubmed | (  ("Public report*"[tiab] OR "public performance report*"[tiab] OR "report card*"[tiab] OR  "comparative performance information"[tiab] OR "consumer report*"[tiab] OR "provider profil*"[tiab] OR  "quality report*"[tiab])  AND  (healthcare[tiab] OR "health care"[tiab] OR hospital*[tiab] OR clinic*[tiab] OR  "health service*"[tiab] OR "health system*"[tiab] OR "medical care"[tiab])  AND  (interoperab*[tiab] OR standard*[tiab] OR HL7[tiab] OR FHIR[tiab] OR  "application programming interface*"[tiab] OR API[tiab] OR "data governance"[tiab] OR  "data quality"[tiab] OR usability[tiab] OR "user experience"[tiab] OR dashboard*[tiab] OR  portal*[tiab] OR architecture[tiab] OR platform*[tiab])  ) |
| 4.2.2026 | Scopus | TITLE-ABS-KEY(  ("Public report*" OR "public performance report*" OR "report card*" OR  "comparative performance information" OR "consumer report*" OR "provider profil*" OR "quality report*")  AND  (healthcare OR "health care" OR hospital* OR clinic* OR "health service*" OR "health system*" OR "medical care")  AND  (interoperab* OR standard* OR HL7 OR FHIR OR "application programming interface*" OR API OR  "data governance" OR "data quality" OR usability OR "user experience" OR dashboard* OR portal* OR  architecture OR platform*)  ) |
| 4.2.2026 | Web of Science | TS=(  ("Public report*" OR "public performance report*" OR "report card*" OR  "comparative performance information" OR "consumer report*" OR "provider profil*" OR "quality report*")  AND  (healthcare OR "health care" OR hospital* OR clinic* OR "health service*" OR "health system*" OR "medical care")  AND  (interoperab* OR standard* OR HL7 OR FHIR OR "application programming interface*" OR API OR  "data governance" OR "data quality" OR usability OR "user experience" OR dashboard* OR portal* OR  architecture OR platform*)  ) |
| 4.2.2026 | ACM | (  ("Public report*" OR "public performance report*" OR "report card*" OR  "comparative performance information" OR "consumer report*" OR "provider profil*" OR "quality report*")  AND  (healthcare OR "health care" OR hospital* OR clinic* OR "health service*" OR "health system*" OR "medical care")  AND  (interoperab* OR standard* OR HL7 OR FHIR OR "application programming interface*" OR API OR  "data governance" OR "data quality" OR usability OR "user experience" OR dashboard* OR portal* OR  architecture OR platform*)  ) |
| 4.2.2026 | IEEE | (  ("Public report*" OR "public performance report*" OR "report card*" OR  "comparative performance information" OR "consumer report*" OR "provider profil*" OR "quality report*")  AND  (healthcare OR "health care" OR hospital* OR clinic* OR "health service*" OR "health system*" OR "medical care")  AND  (interoperab* OR standard* OR HL7 OR FHIR OR "application programming interface*" OR API OR  "data governance" OR "data quality" OR usability OR "user experience" OR dashboard* OR portal* OR  architecture OR platform*)  ) |
| 4.2.2026 | Cochrane | (  ("Public report*" OR "public performance report*" OR "report card*" OR  "comparative performance information" OR "consumer report*" OR "provider profil*" OR "quality report*")  AND  (healthcare OR "health care" OR hospital* OR clinic* OR "health service*" OR "health system*" OR "medical care")  AND  (interoperab* OR standard* OR HL7 OR FHIR OR "application programming interface*" OR API OR  "data governance" OR "data quality" OR usability OR "user experience" OR dashboard* OR portal* OR  architecture OR platform*)  ):ti,ab,kw |
| 4.2.2026 | AIS eLibrary | (  ( "public reporting" OR "public performance report*" OR "report card*" OR "comparative performance information" OR "consumer report*" OR "provider profil*" OR "quality report*" OR "public report*" ) AND ( healthcare OR "health care" OR hospital* OR clinic* OR "health service*" OR "health system*" ) AND (interoperab* OR standard* OR HL7 OR FHIR OR "application programming interface*" OR API OR  "data governance" OR "data quality" OR usability OR "user experience" OR dashboard* OR portal* OR architecture OR platform*)  ) |
