# Supplementary material for: Public Reporting Systems in Health Care and the Underconceptualized Technical Substrate, a Core Information Systems Dimension: Scoping Review
Source: JMIR Med Inform. 2026 Aug 24;14:e84820. doi: 10.2196/84820 (PMC13502770; doi:10.2196/84820)
Supplement: Supplementary file 2 [file medinform-v14-e84820-s004.docx]

**Appendix 3: PRISMA-ScR Checklist**

| Section |  | Item | PRISMA-ScR checklist item | Reported on page # |
| --- | --- | --- | --- | --- |
| Title |  |  |  |  |
| Title |  | 1 | Identify the report as a scoping review | 1 |
| Abstract |  |  |  |  |
| Structured summary |  | 2 | Provide a structured summary that includes: background, aims, eligibility criteria, sources of evidence, charting methods, results and conclusions that relate to the review question and aims. | 1-2 |
| Introduction |  |  |  |  |
| Rationale  Objectives |  | 3  4 | Describe the rationale for the review in the context of what is already known. Explain why the review question lends itself to a scoping review approach  Provide an explicit statement of the questions and objectives being addressed with reference to their key element or other relevant key elements used to conceptualize the review questions and /or objectives | 2-5  2-5 |
| Methods |  |  |  |  |
| Protocol and registration  Eligibility Criteria  Information sources  Search  Selection of sources of evidence  Data charting process  Data items  Critical Appraisal of individual sources of evidence  Synthesis of results |  | 5  6  7  8  9  10  11  12  13 | Indicate whether a review protocol exist; state where it can be accessed, and provide registration information.  Specify characteristics used as eligibility criteria and provide rationale.  Describe all information sources and the date of the most recent search.  Present the full electronic search strategy for at least one database.  State the process for selecting sources of evidence (screening and eligibility).  Describe the methods of charting data and any processes for obtaining and confirming data from investigators.  List and define all variables for which data were sought and any assumptions and simplifications made.  If done, provide a rationale for and methods of critical appraisal of included sources; describe how this information was used.  Describe the methods of handling and summarizing the charted data | 6  7-9  9  9  9  9  9  10  9-10 |
| Results |  |  |  |  |
| Selection of sources of evidence  Characteristics of sources of evidence  Critical appraisal within sources of evidence  Results of individual sources of evidence  Synthesis of results |  | 14  15  16  17  18 | Give numbers of sources of evidence screened, assessed for eligibility, and included in the review, with reasons for exclusions at each stage, ideally using a flow diagram  For each source of evidence, present characteristics for which data were charted and provide the citations.  If done, present data on critical appraisal of included sources of evidence (see item 12)  For each included source of evidence, present the relevant data that were charted that relate to the review questions and objectives.  Summarise and /or present the charting results as they relate to the review questions and objectives. | 10-18  10-18  10-18  10-18  10-18 |
| Discussion |  |  |  |  |
| Summary of evidence  Limitations  Conclusions |  | 19  20  21 | Summarize the main results, link to review questions/objectives, and consider relevance to key groups.  Discuss the limitations of the scoping review process.  Provide a general interpretation of results and potential implications and /or next steps | 18-22  23-24  22-25 |
| Funding |  |  |  |  |
| Funding |  | 23 | Describe sources of funding for included sources of evidence and for the scoping review itself; describe the role of funders. | 26 |
